# Expression and relevance of the G protein-gated K^+^ channel in the mouse ventricle

**DOI:** 10.1038/s41598-018-19719-x

**Published:** 2018-01-19

**Authors:** Allison Anderson, Kanchan Kulkarni, Ezequiel Marron Fernandez de Velasco, Nicholas Carlblom, Zhilian Xia, Atsushi Nakano, Kirill A. Martemyanov, Elena G. Tolkacheva, Kevin Wickman

**Affiliations:** 1 Department of Pharmacology, University of Minnesota, Minneapolis, MN 55455 USA; 2 Department of Biomedical Engineering, University of Minnesota, Minneapolis, MN 55455 USA; 3 Department of Molecular, Cell & Developmental Biology, University of California, Los Angeles, CA 90095 USA; 4 Department of Neuroscience, The Scripps Research Institute, Jupiter, FL 33458 USA

## Abstract

The atrial G protein-gated inwardly rectifying K^+^ (GIRK) channel is a critical mediator of parasympathetic influence on cardiac physiology. Here, we probed the details and relevance of the GIRK channel in mouse ventricle. mRNAs for the atrial GIRK channel subunits (GIRK1, GIRK4), M2 muscarinic receptor (M_2_R), and RGS6, a negative regulator of atrial GIRK-dependent signaling, were detected in mouse ventricle at relatively low levels. The cholinergic agonist carbachol (CCh) activated small GIRK currents in adult wild-type ventricular myocytes that exhibited relatively slow kinetics and low CCh sensitivity; these currents were absent in ventricular myocytes from *Girk1*^−/−^ or *Girk4*^−/−^ mice. While loss of GIRK channels attenuated the CCh-induced shortening of action potential duration and suppression of ventricular myocyte excitability, selective ablation of GIRK channels in ventricle had no effect on heart rate, heart rate variability, or electrocardiogram parameters at baseline or after CCh injection. Additionally, loss of ventricular GIRK channels did not impact susceptibility to ventricular arrhythmias. These data suggest that the mouse ventricular GIRK channel is a GIRK1/GIRK4 heteromer, and show that while it contributes to the cholinergic suppression of ventricular myocyte excitability, this influence does not substantially impact cardiac physiology or ventricular arrhythmogenesis in the mouse.

## Introduction

Increased output of the parasympathetic branch of the autonomic nervous system leads to the slowing of heart rate (HR) and an increase in heart rate variability (HRV), an indicator of beat-to-beat fluctuations in HR^1,2^. Abnormal parasympathetic regulation of cardiac output has been linked to multiple cardiac disorders, including atrial fibrillation (AF), atrioventricular (AV) block, heart failure, and sudden cardiac death^3–7^. The parasympathetic influence on the heart involves acetylcholine (ACh) acting on sino-atrial nodal (SAN) and atrio-ventricular nodal (AVN) cells, as well as atrial myocytes^8–10^. ACh binds to M_2_ muscarinic receptors (M_2_R) on these cells, initiating a branched intracellular signaling pathway mediated by inhibitory (G_i/o_) G proteins, culminating in the activation of the K^+^ channel I_KACh_, as well as the suppression of the cAMP/PKA-dependent cation-selective ion channel (HCN/I_f_) and voltage-gated Ca^2+^ channels^11–14^.

The atrial I_KACh_ channel is a GIRK channel consisting of GIRK1 and GIRK4 subunits, in 1:1 stoichiometry^15,16^. I_KACh_ is gated by G protein Gβγ subunits liberated following M_2_R activation^17,18^. Genetic ablation of either *Girk1* or *Girk4* in mice eliminated the I_KACh_ conductance in neonatal atrial myocytes, and yielded a modest increase in HR and decrease in HRV at baseline in adult animals^19,20^. Additionally, HR and HRV responses to ACh and CCh were diminished in isolated hearts from mice lacking GIRK1 or GIRK4^19–22^.

While the parasympathetic impact on cardiac function is typically ascribed to its influence on the atria, SA node, and AV node, parasympathetic innervation of ventricular tissue is also evident in mammals^10^. In addition, profound effects of vagal nerve stimulation (VNS) on ventricular physiology have been noted in several species^23^. For example, chronic VNS altered the electrophysiological properties of the heart and reduced susceptibility to ventricular arrhythmias in rats^24^. Additionally, ACh shortened action potential duration (APD) in human ventricular myocytes, in an atropine-sensitive manner^25^. This effect has been attributed to the activation of a GIRK channel^23,26–28^. Consistent with this premise, the ACh-induced decrease in APD and effective refractory period (ERP) in rat papillary muscle was blocked by the non-selective GIRK channel blocker tertiapin, and ACh triggered hyperpolarization and a reduction in APD in right ventricle recordings from wild-type but not *Girk4*^−/−^ mice^29^. In addition, a mutation in the human *GIRK4/KCNJ5* gene underlies a congenital form of Long QT Syndrome (LQTS13), a ventricular repolarization disorder associated with arrhythmia, syncope, and sudden death^28,30^.

While available evidence supports the contention that a GIRK channel contributes to the cholinergic influence on ventricular physiology, critical details remain unclear. Here, we examined the expression in mouse ventricle of genes implicated in atrial I_KACh_-dependent signaling, and evaluated the impact of gene ablation on cholinergic signaling in ventricular myocytes. We present functional evidence that the GIRK channel in ventricular myocytes is a GIRK1/GIRK4 heteromer, and that it mediates the impact of cholinergic signaling on APD and excitability of these cells. We also probed the physiological impact of ventricular GIRK-dependent signaling using a novel ventricle-specific *Girk1*^−/−^ mouse line. Our findings reveal that, despite the contribution of GIRK channels to the CCh-dependent acceleration of repolarization and decrease in ventricular myocyte excitability, ventricular GIRK-dependent signaling does not exert a significant impact on HR, HRV, or susceptibility to ventricular arrhythmias in mice.

## Results

### Expression of I_KACh_-dependent signaling pathway elements in mouse atria and ventricle

**Figure 1:**
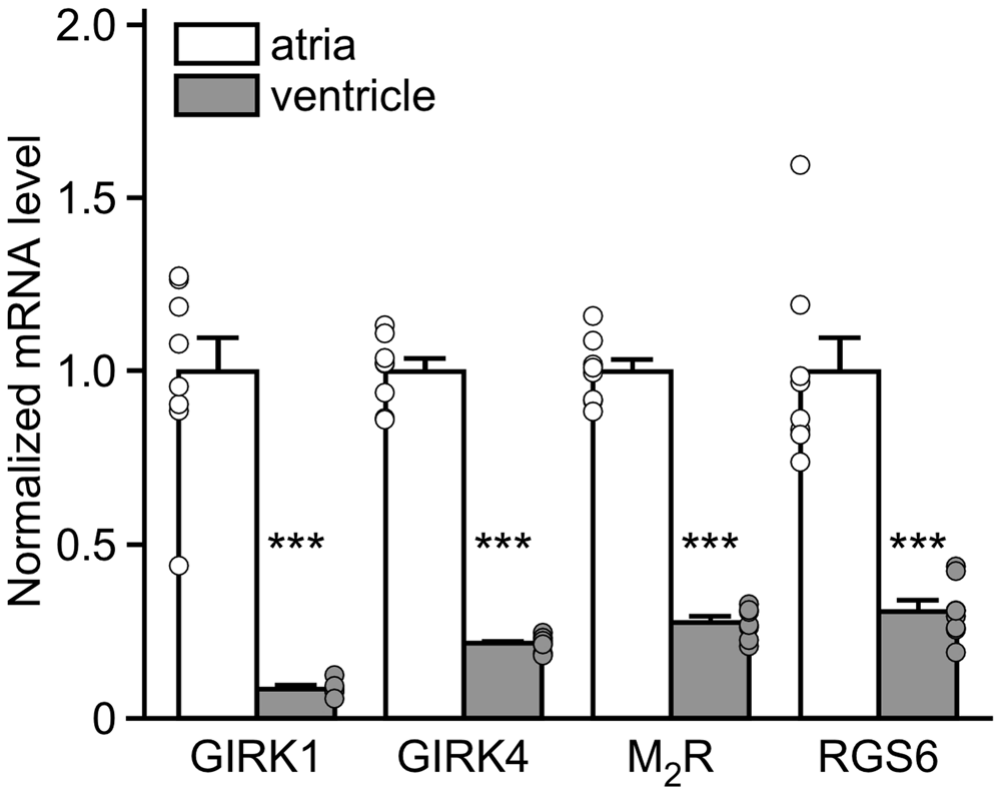
Expression of I_KACh_-dependent signaling elements in mouse atria and ventricle. mRNA levels of GIRK1 (*t*_14_ = 9.4, ***P < 0.001), GIRK4 (*t*_14_ = 21.2, ***P < 0.001), M_2_R (*t*_13_ = 18.6, ***P < 0.001), and RGS6 (*t*_14_ = 6.7, ***P < 0.001), and in atria and ventricle from adult C57BL/6J mice, compared using an unpaired Student’s t-test. mRNA levels of each target were normalized to the level of GAPDH mRNA measured in each sample. Ventricular mRNA levels for each target was normalized to the level present in atrial samples (n = 7–8 samples per tissue, per target).

Expression of key elements of the atrial M_2_R-I_KACh_ signaling pathway, including GIRK1, GIRK4, M_2_R, and RGS6, has been detected in ventricular tissue from many mammalian species^23,26,28,31–33^. A study in the rat heart revealed higher expression in atria than ventricle for GIRK1, GIRK4, and RGS6^34^. We sought to confirm these findings in the mouse by comparing the expression levels of these targets, as well as M_2_R, in the atria and ventricle of adult mice using quantitative RT-PCR. We found that GIRK1, GIRK4, M_2_R, and RGS6 mRNAs were all present in mouse ventricular tissue, but that the levels of all targets were lower (4- to 10-fold) than those found in mouse atrial tissue (Fig. 1).

### Evidence of a GIRK1/GIRK4 heteromer in mouse ventricular myocytes

Previous work suggests that a GIRK4-containing GIRK channel is present in rodent ventricle^29,35^. While GIRK1/GIRK4 heteromeric channels are thought to mediate the atrial I_KACh_ conductance, some evidence suggests that GIRK4 homomeric channels may contribute as well^36,37^. To assess the relative contributions of GIRK1 and GIRK4 to the GIRK channel activity in ventricle, we measured whole-cell currents induced by the non-selective cholinergic agonist carbachol (CCh) in ventricular myocytes from wild-type mice, and from mice lacking GIRK1 or GIRK4. Ventricular myocytes express a prominent constitutive K^+^ current (I_K1_) that masks the ACh/CCh-induced GIRK current under normal whole-cell recording conditions, due to K^+^ accumulation and/or depletion^38^. As such, we included a low concentration of Ba^2+^ (5 μM) in our bath solution to selectively suppress I_K1_, while preserving any potential GIRK channel activity. Indeed, 5 μM Ba^2+^ did not suppress the CCh-induced GIRK current in wild-type SAN cells; indeed, currents were slightly larger in the presence Ba^2+^ (Supplementary Fig. S1).

**Figure 2:**
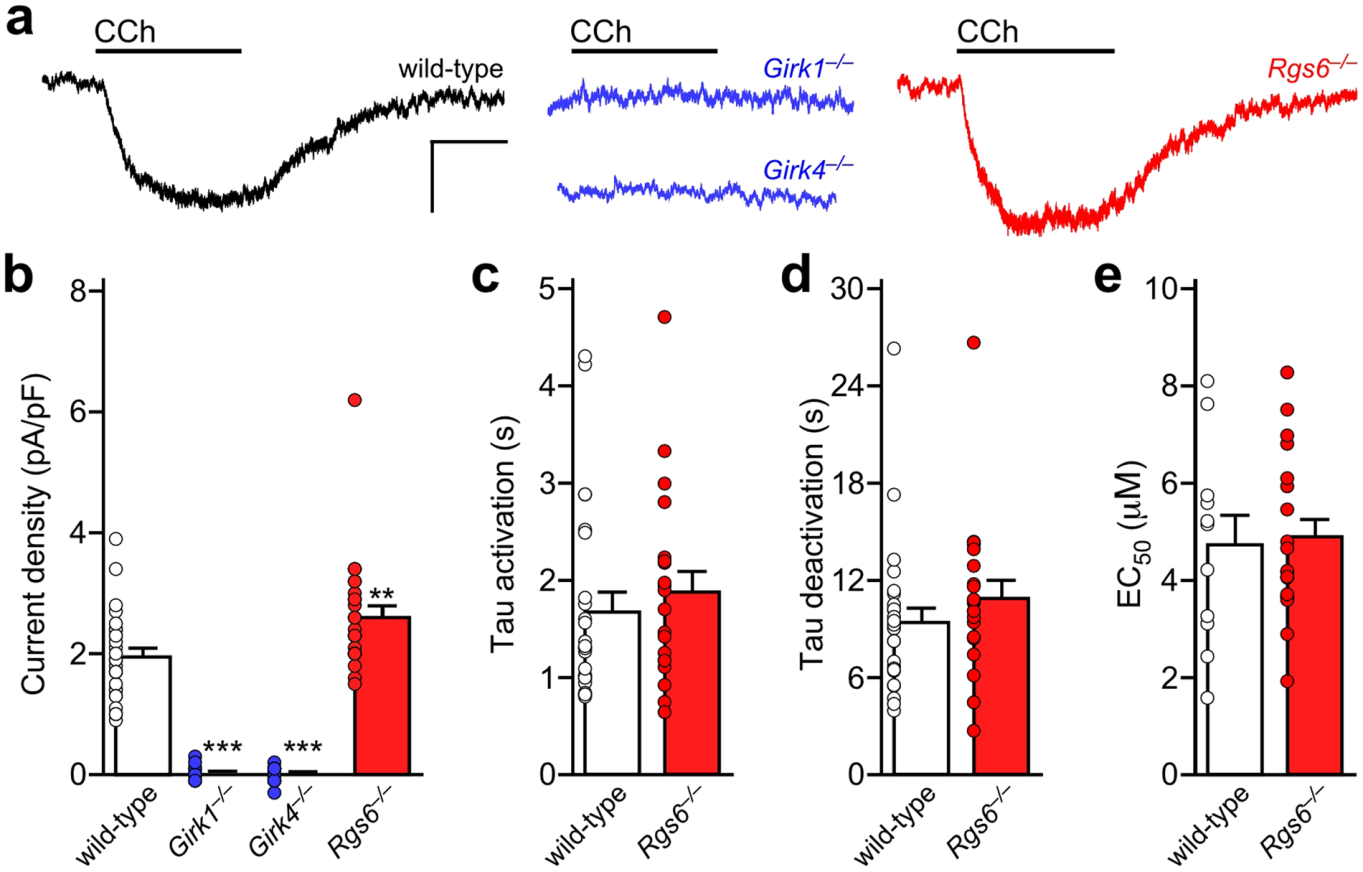
Carbachol-induced GIRK currents in adult mouse ventricular myocytes. (**a**) Whole-cell currents (V_hold_ = −70 mV) evoked by carbachol (CCh, 100 μM) in a high-K^+^ bath solution (containing 5 μM BaCl_2_) in adult ventricular myocytes from wild-type, *Girk1*^−/−^, *Girk4*^−/−^, and *Rgs6*^−/−^ mice. Scale: 0.1 nA/10 s. (**b**) Summary of maximal CCh-induced current density in adult ventricular myocytes from wild-type (n = 28 cells/6 mice), *Girk1*^−/−^ (n = 23 cells/4 mice), *Girk4*^−/−^ (n = 21 cells/4 mice), and *Rgs6*^−/−^ mice (n = 24 cells/6 mice); open symbols overlapping the bars denote individual data points. One-way ANOVA analysis revealed an effect of genotype on CCh-induced current density (F_3,92_ = 110.6, *P* < 0.001). Symbols: **^,^ ***P < 0.01 and 0.001, respectively, vs. wild-type. (**c**,**d**) Summary of activation (c; *t*_44_ = 0.7, *P* = 0.51) and deactivation (**d**); *t*_44_ = 1.1, *P* = 0.28) kinetics for the CCh-induced current in ventricular myocytes from wild-type (n = 25 cells/6 mice) and *Rgs6*^−/−^ mice (n = 21 cells/6 mice), compared across genotypes using an unpaired Student’s t-test. (**e**) Summary of EC_50_ values derived from concentration-response experiments for the CCh-induced current in adult ventricular myocytes from wild-type vs. *Rgs6*^−/−^ mice. The EC_50_ for current activation by CCh did not differ between wild-type (4.8 ± 0.6 μM, n = 11 cells/4 mice) and *Rgs6*^−/−^ (4.9 ± 0.4 μM, n = 19 cells/4 mice) ventricular myocytes, as determined using an unpaired Student’s t-test (*t*_28_ = 0.20, *P* = 0.84).

CCh (100 μM) evoked reliable currents in ventricular myocytes from adult wild-type mice, but not in myocytes from *Girk1*^−/−^ or *Girk4*^−/−^ mice (Fig. 2a,b). Since GIRK1 is unable to form functional homomeric channels^15,39–42^, this observation supports the contention that GIRK1 and GIRK4 assemble to form the GIRK channel in mouse ventricular myocytes. The peak density of the CCh-induced GIRK current was ~5–10 fold smaller in ventricular myocytes, however, as compared to currents reported in SAN cells^43^. Additionally, the CCh-induced current activation and deactivation kinetics were significantly slower for the ventricular GIRK channel as compared to the atrial GIRK channel (Fig. 2c,d)^43^. Furthermore, the ventricular GIRK current was more than an order of magnitude less sensitive to CCh (EC_50_ = 4.8 ± 0.6 μM; Fig. 2e) than the SAN cell GIRK current (EC_50_ = 0.25 ± 0.03 μM)^43^, reminiscent of the lower ACh sensitivity of the GIRK conductance in human ventricular myocytes as compared to atrial myocytes^44^.

### Influence of RGS6 on GIRK-dependent signaling in ventricular myocytes

The M_2_R-I_KACh_ signaling pathway in atrial myocytes and SAN cells is negatively regulated by Regulator of G protein Signaling 6 (RGS6)^22,32,43,45^. Indeed, in SAN cells and atrial myocytes from mice lacking RGS6, GIRK currents evoked by CCh exhibited slower deactivation kinetics and an increased sensitivity to CCh^32,43^. To test whether ventricular GIRK-dependent signaling is negatively regulated by RGS6, we compared CCh-induced GIRK currents in ventricular myocytes from adult wild-type and *Rgs6*^−/−^ mice. Current densities were slightly, but significantly, larger in ventricular myocytes from *Rgs6*^−/−^ mice as compared to wild-type controls (Fig. 2a,b). No effect of *Rgs6* ablation, however, was seen on activation (Fig. 2c) or deactivation (Fig. 2d) kinetics of the CCh-induced, ventricular GIRK current. Moreover, the EC_50_ for CCh-induced activation of the GIRK current was comparable in ventricular myocytes from wild-type and *Rgs6*^−/−^ mice (Fig. 2e). Thus, the impact of RGS6 on ventricular GIRK-dependent signaling is limited to a modest negative regulation of peak current density.

### Influence of GIRK channels on ventricular myocyte APD and excitability

**Figure 3:**
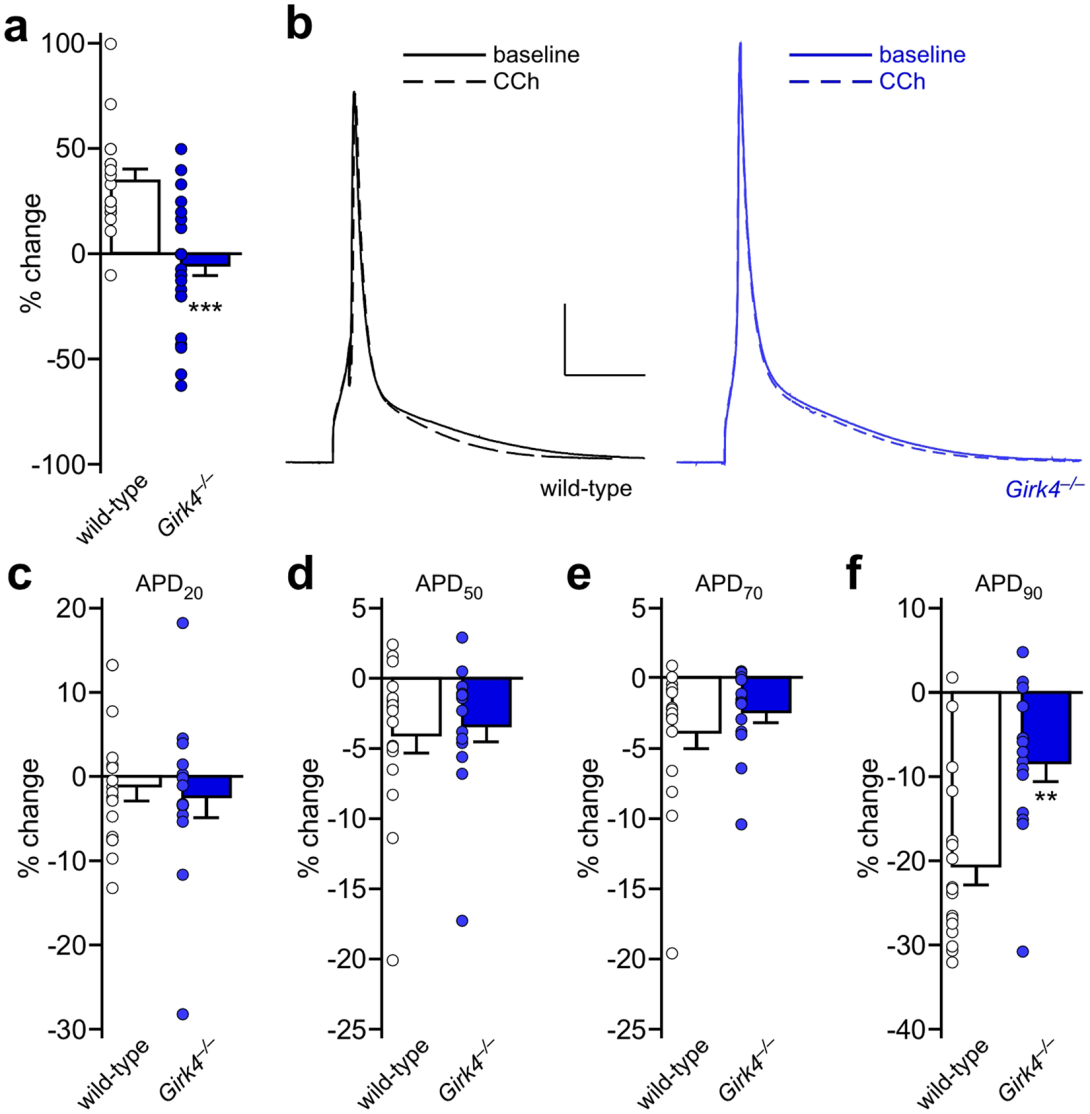
GIRK channel contribution to the cholinergic regulation of mouse ventricular myocyte repolarization and excitability. (**a**) Change in rheobase evoked by CCh (100 μM) in adult wild-type (n = 19 cells/5 mice) and *Girk4*^−/−^ (n = 26 cells/6 mice) ventricular myocytes, compared using an unpaired Student’s t-test (*t*_43_ = 5.1, ***P < 0.001). (**b**) Action potentials evoked by current injection in wild-type and *Girk4*^−/−^ ventricular myocytes, at baseline (solid line) and in the presence of CCh (100 μM, dashed line). Scale: 20 mV/25 ms. (**c**–**f**) Summary of the percentage change in APD_20_ (*t*_29_ = 0.37, *P* = 0.72), APD_50_ (*t*_29_ = 0.38, *P* = 0.71), APD_70_ (*t*_29_ = 0.97, *P* = 0.34), and APD_90_ (*t*_29_ = 3.5, **P < 0.01) evoked by CCh in wild-type (n = 17 cells/6 mice) and *Girk4*^−/−^ (n = 14 cells/5 mice) ventricular myocytes, compared using an unpaired Student’s t-test.

To test whether I_KACh_ modulates ventricular myocyte excitability and repolarization, we evaluated the change in rheobase and APD induced by CCh (100 μM) in ventricular myocytes from wild-type and *Girk4*^−/−^ mice. The CCh-induced decrease in ventricular myocyte excitability (increase in rheobase) was larger in ventricular myocytes from wild-type than *Girk4*^−/−^ mice (Fig. 3a). And, although there was no significant difference in the CCh-induced shortening of APD_20_, APD_50_, and APD_70_ between ventricular myocytes from wild-type and *Girk4*^−/−^ mice (Fig. 3b–e), the CCh-induced decrease in APD_90_ was blunted in ventricular myocytes from *Girk4*^−/−^ mice (Fig. 3f). Thus, GIRK channels contribute to the cholinergic regulation of excitability and repolarization of adult mouse ventricular myocytes.

### Generation and characterization of mice lacking GIRK channels in ventricle

**Figure 4:**
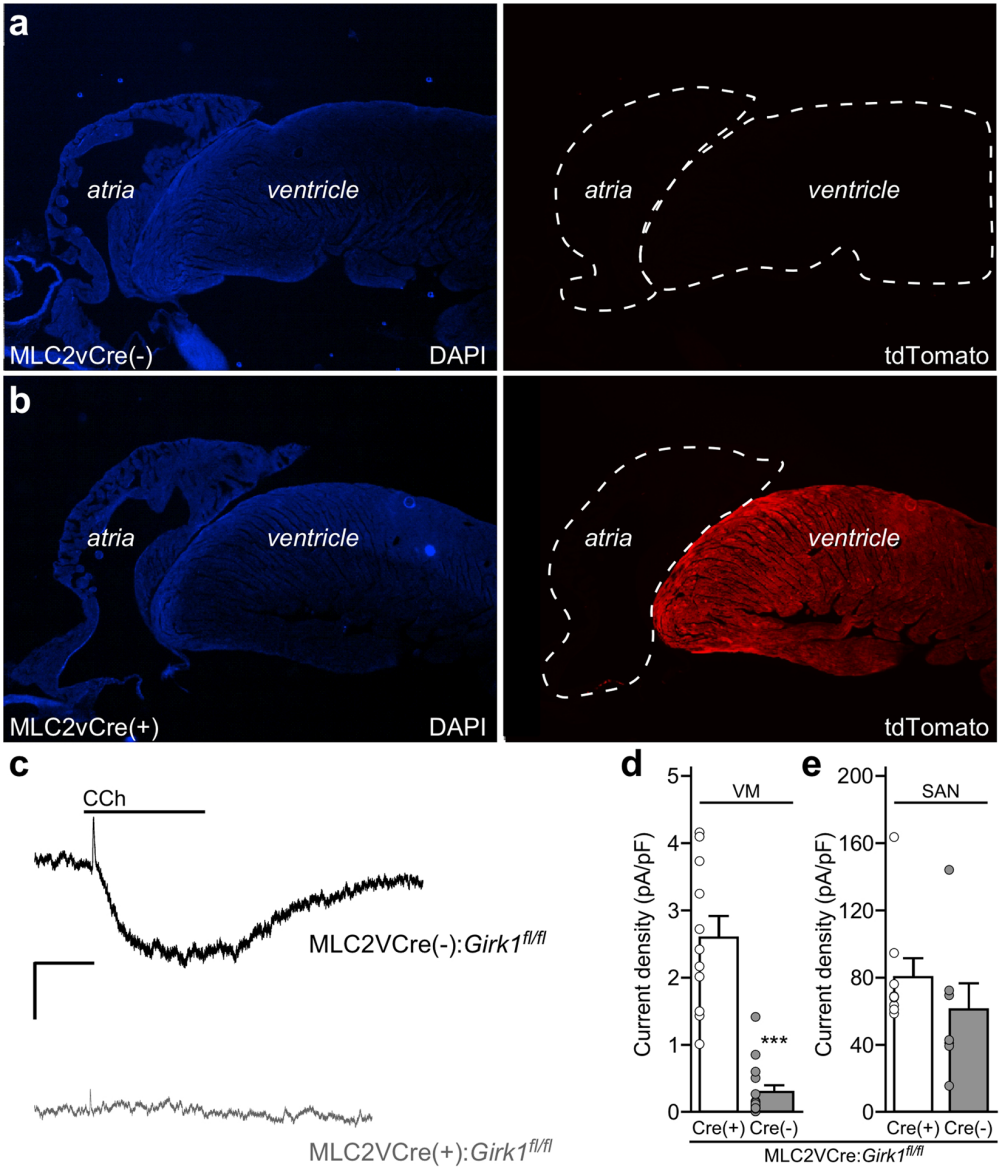
Characterization of mice lacking GIRK channels in the ventricle. (**a**,**b**) Sections of the heart from MLC2VCre(−):Ai14-tdTomato and MLC2VCre(+):Ai14-tdTomato mice, labeled with the nuclear stain DAPI (left panels), showing restricted Cre-dependent gene expression (tdTomato, right panels) in the ventricle of MLC2VCre(+) mice. (**c**) Whole-cell currents (V_hold_ = − 70 mV) evoked by CCh (100 μM) in a high-K^+^ bath solution (containing 5 μM BaCl_2_) in adult ventricular myocytes from MLC2VCre(−):*Girk1*^*fl/fl*^ and MLC2VCre(+):*Girk1*^*fl/fl*^ mice. Scale: 0.2 nA/10 s. (**d**) Summary of CCh-induced current densities in MLC2VCre(−):*Girk1*^*fl/fl*^ (n =1 1 cells/2 mice) and MLC2VCre(+):*Girk1*^*fl/fl*^ (n = 15 cells/2 mice) ventricular myocytes (VM), compared with an unpaired Student’s t-test (*t*_24_ = 7.5, ***P < 0.001). (**e**) Summary of CCh-induced current densities in adult SAN cells from MLC2VCre(−):*Girk1*^*fl/fl*^ (n = 9 cells/3 mice) and MLC2VCre(+):*Girk1*^*fl/fl*^ (n = 7 cells/2 mice) mice, compared with an unpaired Student’s t-test (*t*_14_ = 1.1, *P* = 0.31).

To probe the impact of ventricular GIRK channel on cardiac physiology, we crossed a conditional *Girk1* knockout mouse (*Girk1*^*fl/fl*^)^46^ with a ventricle-specific Cre driver line (MLC2VCre)^47,48^. We first verified the ventricular specificity of Cre-dependent gene ablation conferred by the MLC2VCre driver line using a Cre-dependent fluorescent reporter mouse line (Fig. 4a,b). We next evaluated the functional impact and cell specificity of *Girk1* ablation by measuring CCh-induced whole-cell currents in SAN cells and ventricular myocytes from MLC2VCre(−):*Girk1*^*fl/fl*^ and MLC2VCre(+):*Girk1*^*fl/fl*^ mice. CCh-induced current density in adult ventricular myocytes from MLC2VCre(+):*Girk1*^*fl/fl*^ mice was significantly smaller than responses measured in ventricular myocytes from MLC2VCre(−):*Girk1*^*fl/fl*^ littermates (Fig. 4c,d). We observed no difference, however, in CCh-induced current density in SAN cells from adult MLC2VCre(+):*Girk1*^*fl/fl*^ and MLC2VCre(−):*Girk1*^*fl/fl*^ mice (Fig. 4e). Thus, GIRK channel activity is selectively suppressed in ventricular myocytes from MLC2VCre(+):*Girk1*^*fl/fl*^ mice.

### Impact of ventricle-specific GIRK channel ablation on cardiac physiology

**Figure 5:**
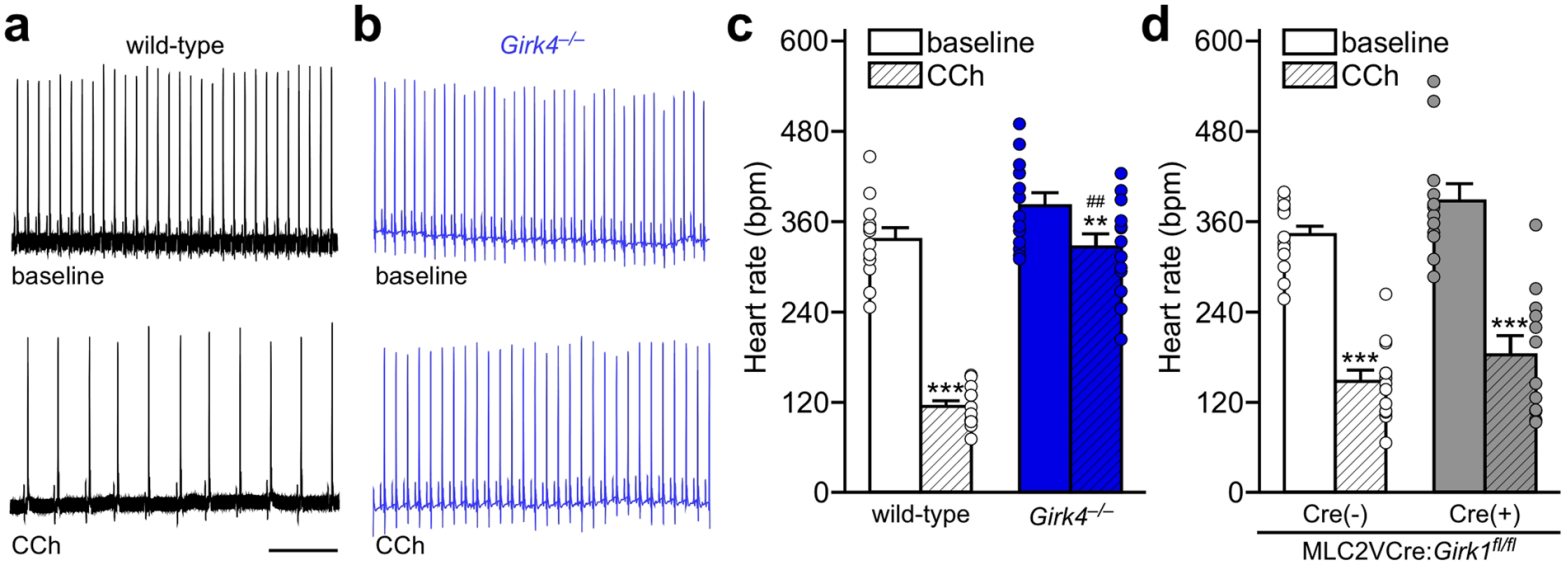
HR analysis of constitutive and ventricle-specific *Girk*^−/−^ mice. (**a**,**b**) Segments of ECG recordings from anesthetized wild-type (**a**) and *Girk4*^−/−^ (**b**) mice, at baseline and after injection of CCh (1.0 mg/kg i.p.). Scale: 1 s. (**c**) Summary of HR data at baseline and after injection of CCh for wild-type (n = 12) and *Girk4*^−/−^ (n = 13) mice. Two-way ANOVA analysis revealed an interaction between genotype and treatment (F_1,23_ = 54.8, *P* < 0.001). Symbols: **^,^***P < 0.01 and 0.001, respectively, vs. baseline (within genotype); ^##^*P* < 0.01 vs. wild-type (within treatment). (**d**) Summary of HR data at baseline and after injection of CCh for MLC2VCre(+):*Girk1*^*fl/fl*^ (n = 13) and MLC2VCre(−):*Girk1*^*fl/fl*^ (n = 12) littermates. There was a significant main effect of treatment (F_1,23_ = 173.1, *P* < 0.001), but no main effect of genotype (F_1,23_ = 3.6, *P* = 0.070), or interaction between genotype and treatment (F_1,23_ = 0.09, *P* = 0.77). Symbols: ***P < 0.001 vs. baseline (within genotype).

**Figure 6:**
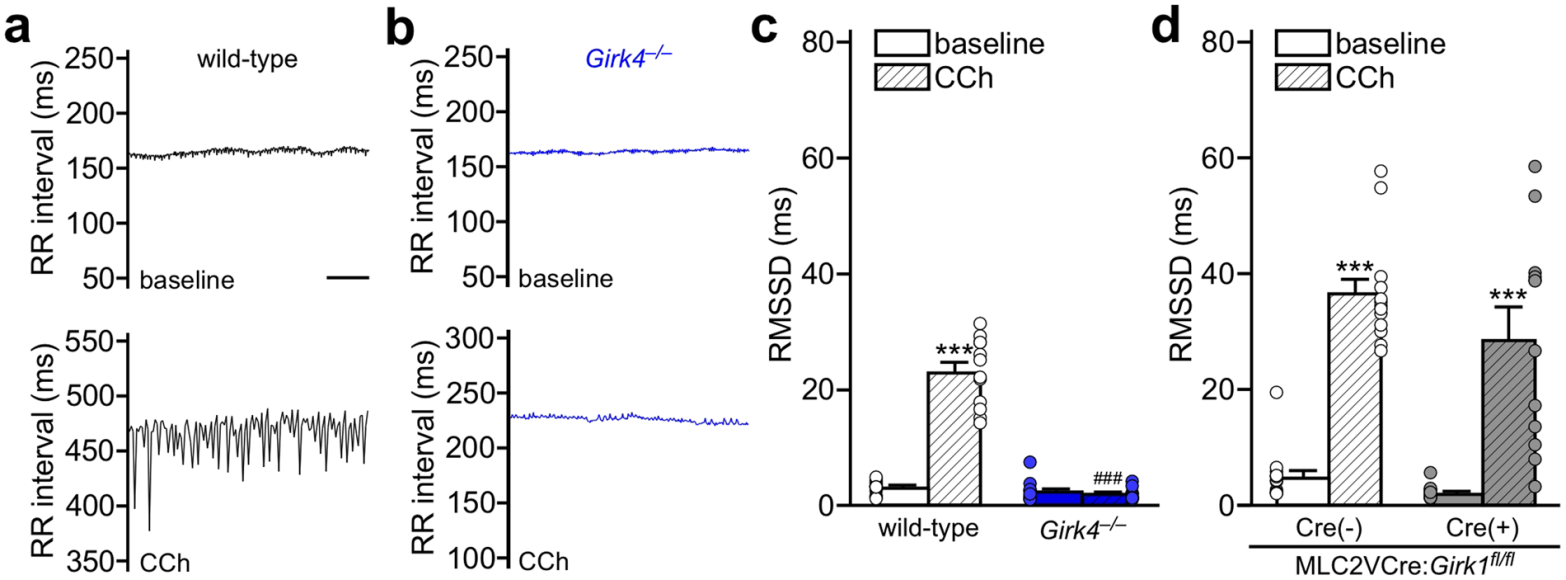
Time-domain HRV analysis of constitutive and ventricle-specific *Girk*^−/−^ mice. (**a**,**b**) RR tachograms for anesthetized wild-type (**a**) and *Girk4*^−/−^ (**b**) mice, at baseline and following injection of CCh (1.0 mg/kg i.p.). Scale: 10 s. (**c**) Summary of RMSSD data at baseline and following injection of CCh for wild-type (n = 12) and *Girk4*^−/−^ (n=13) mice. Two-way ANOVA analysis revealed an interaction between genotype and treatment (F_1,23_ = 158.7, *P* < 0.001). Symbols: ***P < 0.001 vs. baseline (within genotype); ^###^*P* < 0.001 vs. wild-type (within treatment). (**d**) Summary of RMSSD data at baseline and following injection of CCh for MLC2VCre(−):*Girk1*^*fl/fl*^ (n = 13) and MLC2VCre(+):*Girk1*^*fl/fl*^ (n = 12) mice. There was a significant main effect of treatment (F_1,23_ = 96.3, *P* < 0.001), but no main effect of genotype (F_1,23_ = 3.2, *P* = 0.087) or interaction between genotype and treatment (F_1,23_ = 0.66, *P* = 0.43). Symbols: ***P < 0.001 vs. baseline (within genotype).

**Figure 7:**
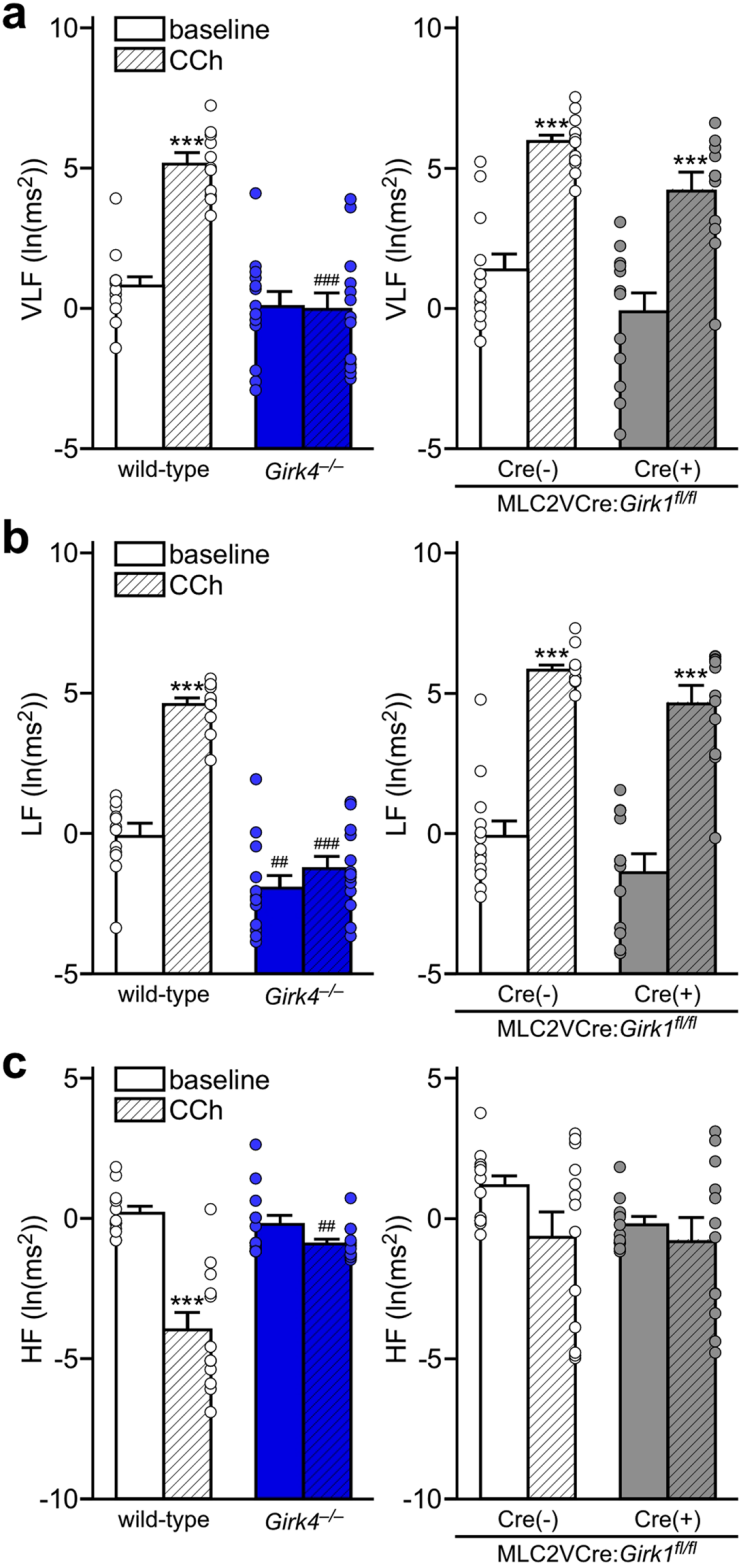
Frequency domain HRV analysis in constitutive and ventricle-specific *Girk*^−/−^ mice. (**a**) Power in the VLF ( < 0.4 Hz) range at baseline and following injection of CCh (1.0 mg/kg i.p.). Two-way ANOVA analysis revealed an interaction between genotype and treatment (F_1,23_ = 36.3, *P* < 0.001) for wild-type (n = 12) and *Girk4*^−/−^ (n = 13) mice (left panel). While main effects of treatment (F_1,23_ = 51.0, *P* < 0.001) and genotype (F_1,23_ = 13.77, *P* < 0.01) were observed for MLC2VCre(−):*Girk1*^*fl/fl*^ (n = 13) and MLC2VCre(+):*Girk1*^*fl/fl*^ (n = 12) mice (right panel), there was no interaction between genotype and treatment (F_1,23_ = 0.01, *P* = 0.90). Symbols: ***P < 0.001 vs. baseline (within genotype); ^###^*P* < 0.001 vs. wild-type (within treatment). (**b**) Power in the LF (0.4–1.5 Hz) range at baseline and following injection of CCh (1.0 mg/kg i.p.). Two-way ANOVA analysis revealed an interaction between genotype and treatment (F_1,23_ = 41.6, *P* < 0.001) for wild-type and *Girk4*^−/−^ mice (left panel). While main effects of treatment (F_1,23_ = 113.1, *P* < 0.001) and genotype (F_1,23_ = 8.8, *P* < 0.01) were observed for MLC2VCre(−):*Girk1*^*fl/fl*^ and MLC2VCre(+):*Girk1*^*fl/fl*^ mice (right panel), there was no interaction between genotype and treatment (F_1,23_ = 0.02, *P* = 0.88). Symbols: ***P < 0.001 vs. baseline (within genotype); ^##,###^*P* < 0.01 and 0.001, respectively, vs. wild-type (within treatment). (**c**) Power in the HF (1.5–5.0 Hz) range at baseline and following injection of CCh (1.0 mg/kg i.p.). Two-way ANOVA analysis revealed an interaction between genotype and treatment (F_1,23_ = 37.5, *P* < 0.001) for wild-type and *Girk4*^−/−^ mice (left panel). There were no main effects of treatment (F_1,23_ = 2.7, *P* = 0.11) or genotype (F_1,23_ = 1.8, *P* = 0.19) for MLC2VCre(−):*Girk1*^*fl/fl*^ and MLC2VCre(+):*Girk1*^*fl/fl*^ mice (right panel), nor was there an interaction between genotype and treatment (F_1,23_ = 0.8, *P* = 0.38). Symbols: ***P < 0.001 vs. baseline (within genotype). ^##^*P* < 0.01 vs. wild-type (within treatment).

To discern the impact of ventricle-specific GIRK channel ablation on cardiac physiology, we evaluated electrocardiogram (ECG) recordings from anesthetized MLC2VCre(+):*Girk1*^*fl/fl*^ and MLC2VCre(−):*Girk1*^*fl/fl*^ mice, as well as wild-type and constitutive *Girk4*^−/−^ mice. We measured HR (Fig. 5) and HRV (Figs 6 and 7), as well as specific waveform parameters (PR, QT, and QTc intervals; Supplementary Table 1), at baseline and following systemic administration of CCh (1.0 mg/kg i.p.). At baseline, HR was slightly higher in *Girk4*^−/−^ mice relative to wild-type controls, but this difference did not reach the level of statistical significance (Fig. 5a–c). Similarly, no significant difference in baseline HR was observed in MLC2VCre(+):*Girk1*^*fl/fl*^ mice relative to MLC2VCre(−):*Girk1*^*fl/fl*^ controls (Fig. 5d). As expected, CCh decreased HR in wild-type mice, and this effect was significantly smaller in *Girk4*^−/−^ mice (Fig. 5a–c). In contrast, CCh-induced bradycardia was comparable in MLC2VCre(+):*Girk1*^*fl/fl*^ and MLC2VCre(−):*Girk1*^*fl/fl*^ mice (Fig. 5d). Arrhythmic events, defined as instances of AV block or tachycardic episodes, were also quantified before and after CCh injection (Supplementary Fig. S2). While no arrhythmic events were observed at baseline for any genotype, wild-type mice exhibited more arrhythmic events than *Girk4*^−/−^ mice after CCh injection. Both MLC2VCre(+):*Girk1*^*fl/fl*^ mice and MLC2VCre(−):*Girk1*^*fl/fl*^ mice exhibited a similar frequency of arrhythmic events after CCh injection.

We next evaluated HRV, examining the root mean square of successive differences (RMSSD) in R-R interval before and after CCh injection. While baseline RMSSD in *Girk4*^−/−^ mice was slightly lower in comparison to wild-type mice, this difference did not reach the level of statistical significance (Fig. 6a–c). Consistent with published data^22^, the CCh-induced increased in RMSSD was significantly blunted in *Girk4*^−/−^ mice relative to wild-type controls (Fig. 6c). MLC2VCre(+):*Girk1*^*fl/fl*^ and MLC2VCre(−):*Girk1*^*fl/fl*^ mice displayed comparable RMSSD values, both prior to and after CCh injection (Fig. 6c).

Frequency domain analysis can offer a more sensitive measure of the relative contributions of the sympathetic and parasympathetic branches of the autonomic nervous system to HRV^49^. While power in the VLF range (<0.4 Hz) at baseline did not differ between wild-type and *Girk4*^−/−^ mice, CCh increased VLF power in wild-type but not *Girk4*^−/−^ mice (Fig. 7a, left). In the LF range (0.4–1.5 Hz), *Girk4*^−/−^ mice exhibited reduced power relative to wild-type controls at baseline and, in contrast to wild-type mice, a lack of increase induced by CCh (Fig. 7b, left). In the HF range (1.5–5.0 Hz), we observed no difference between wild-type and *Girk4*^−/−^ mice at baseline. Interestingly, CCh administration decreased power in this frequency range in wild-type mice, but not *Girk4*^−/−^ mice (Fig. 7c, left). No difference in power in VLF, LF, and HF domains was observed between MLC2VCre(+):*Girk1*^*fl/fl*^ and MLC2VCre(−):*Girk1*^*fl/fl*^ mice, either at baseline or after CCh administration (Fig. 7a–c, right).

With respect to key parameters of the ECG waveform (Supplementary Table 1), no significant genotype differences were observed for PR and QT intervals at baseline. CCh significantly prolonged both the PR and QT intervals in wild-type mice, and this effect was blunted in *Girk4*^−/−^ mice. PR and QT intervals were also prolonged in MLC2VCre(+):*Girk1*^*fl/fl*^ and MLC2VCre(−):*Girk1*^*fl/fl*^ mice after CCh administration, but there was no difference between genotypes. We also evaluated the corrected QT (QTc) interval, a measure that can account for the influence of HR on the QT interval^50^, using a variation of the human QT correction formula that assumes a typical RR interval for a conscious mouse under normal conditions (100 ms). We observed no difference in QTc interval between wild-type and *Girk4*^−/−^ mice, or MLC2VCre(+):*Girk1*^*fl/fl*^ or MLC2VCre(−):*Girk1*^*fl/fl*^ mice, at baseline. Interestingly, while CCh administration had no effect on QTc interval in wild-type mice, it lengthened this interval in *Girk4*^−/−^ mice. QTc interval was not prolonged after CCh injection, however, in either MLC2VCre(+):*Girk1*^*fl/fl*^ or MLC2VCre(−):*Girk1*^*fl/fl*^ mice.

### Impact of ventricle-specific GIRK channel ablation on pacing-induced ventricular arrhythmia

**Figure 8:**
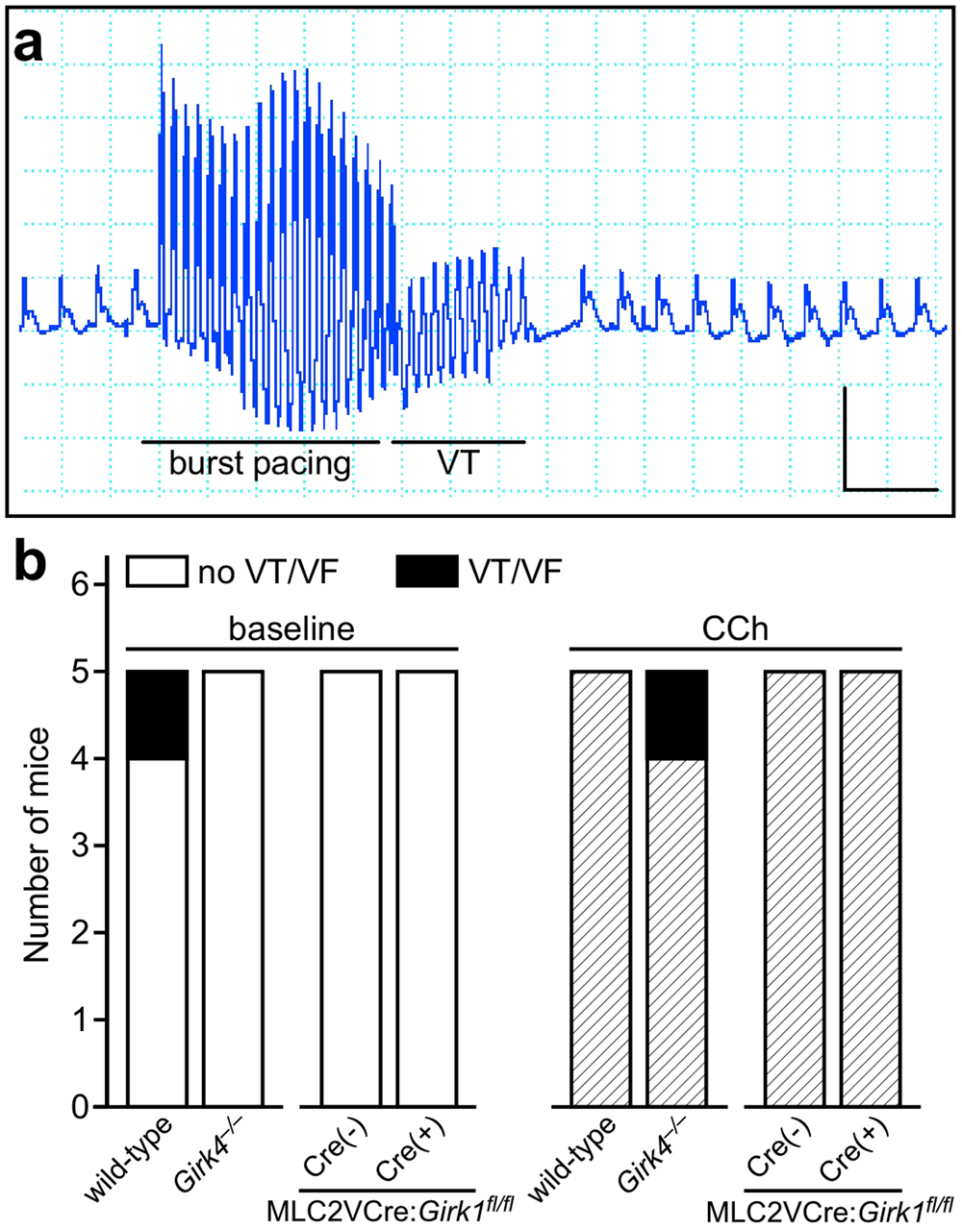
Susceptibility to pacing-induced ventricular arrhythmias in constitutive and ventricle-specific *Girk*^−/−^ mice. (**a**) Recording of a VT episode after infusion of CCh (3 μM) and burst pacing of the left ventricle. Scale: 0.5 V/0.2 s. (**b**) Summary of the number of mice exhibiting VT/VF episodes after burst pacing of the left ventricle at baseline (left) and after CCh perfusion (right). Fisher’s exact test revealed no differences in the occurrence of VT/VF episodes between wild-type and *Girk4*^−/−^ mice, either at baseline (*P* = 1.0) or after CCh administration (*P* = 1.0). We did not observe any incidents of VT/VF in MLC2VCre(−):*Girk1*^*fl/fl*^ or MLC2VCre(+):*Girk1*^*fl/fl*^ mice, either at baseline or after CCh administration.

To investigate the contribution of the ventricular GIRK channel to ventricular arrhythmogenesis, isolated hearts from MLC2VCre(+):*Girk1*^*fl/fl*^ and MLC2VCre(−):*Girk1*^*fl/fl*^ mice, as well as wild-type and constitutive *Girk4*^−/−^ mice, were subjected to burst pacing in the absence and presence of CCh to discern differences in susceptibility to ventricular tachycardia (VT) or fibrillation (VF) (Fig. 8a). There was no significant difference in the incidence of pacing-induced VT/VF at baseline between wild-type and *Girk4*^−/−^ hearts (Fig. 8b, left). Similarly, hearts from MLC2VCre(+):*Girk1*^*fl/fl*^ and MLC2VCre(−):*Girk1*^*fl/fl*^ mice did not differ in the incidence of VT/VF in response to pacing stimuli at baseline. After CCh perfusion, there was, again, no significant difference in incidence of pacing-induce VT/VF between wild-type and *Girk4*^−/−^ hearts (Fig. 8b, right). Likewise, no difference was observed in the incidence of pacing-induced VT/VF between MLC2VCre(+):*Girk1*^*fl/fl*^ and MLC2VCre(−):*Girk1*^*fl/fl*^ hearts. Collectively, these observations suggest that loss of the ventricular GIRK channel does not impact susceptibility to pacing-induced ventricular arrhythmias.

## Discussion

In this study, we present evidence that that GIRK channel found in adult mouse ventricular myocytes is, like the channel described previously in atrial myocytes and SAN cells, a GIRK1/GIRK4 heteromultimeric channel. Nevertheless, the GIRK current in ventricular myocytes exhibited several properties that differed from the GIRK current described previously in atrial myocytes and SAN cells^43,45^. Notably, the ventricular GIRK current was smaller and less sensitive to CCh. The differences in GIRK conductance between atrial and ventricular myocytes could be due to increased expression and/or influence of a negative regulator of GIRK-dependent signaling in ventricle. Our data suggest that increased ventricular expression or influence of RGS6, however, does not explain the functional differences between the mouse atrial and ventricular GIRK conductance. Indeed, RGS6 mRNA levels were substantially lower in ventricle than in atria. Moreover, *Rgs6* ablation did not enhance the CCh sensitivity or alter the kinetics of the ventricular GIRK current. It is possible that other RGS proteins account for the differences observed between in atrial and ventricular GIRK-dependent signaling in the mouse heart. Alternatively, elements of the GIRK-dependent signaling pathway may be compartmentalized in distinct fashion in the atria and ventricle, or additional positive and/or negative regulatory elements that impact I_KACh_-dependent signaling remain to be discovered.

A recent study reported that the ACh-induced shortening of ventricular APD_90_ was diminished in isolated hearts from *Girk4*^−/−^ mice, suggesting that a GIRK4-containing GIRK channel facilitates ventricular repolarization in the mouse heart^29^. Consistent with this premise, we observed a significant impact of *Girk4* ablation on the CCh-induced shortening of APD_90_ in isolated ventricular myocytes from adult mice. Our efforts in isolated ventricular myocytes also revealed that *Girk4* ablation attenuates the CCh-induced decrease in ventricular myocyte excitability. These results support our contention that the GIRK channel in ventricular myocytes contributes to the cholinergic regulation of ventricular myocyte APD and excitability.

I_KACh_ is a critical downstream mediator of parasympathetic effects on atrial physiology and HR regulation. Previous studies with ECG telemetry and isolated mouse hearts have shown that a loss of I_KACh_ correlates with altered baseline measures and diminished responses to pharmacologic manipulations that enhance or mimic parasympathetic input to the heart^19–22^. Here, we replicated several reported observations in *Girk4*^−/−^ mice, including the attenuation of CCh-induced bradycardia. While we did not see a significant baseline tachycardia in *Girk4*^−/−^ mice in this study, this may reflect an influence of anesthesia. We additionally observed evidence of diminished HRV at baseline (LF), and after CCh administration (RMSSD, VLF, and LF), in *Girk4*^−/−^ mice. Our parallel evaluation of mice lacking GIRK channels selectively in ventricle indicate that most, if not all, of the cardiac phenotypes reported in constitutive *Girk1*^−/−^ or *Girk4*^−/−^ mice are attributable to a loss of atrial I_KACh_. Surprisingly, we found that CCh decreased power in the HF domain in wild-type mice, but not in *Girk4*^−/−^ mice. As fluctuations in HR due to respiration contribute to power in the HF range^51^, our observation could reflect a contribution of a GIRK4-containing GIRK channel to the cholinergic regulation of respiration. The CCh-induced decrease in HF power was not significant in MLC2VCre(−):*Girk1*^*fl/fl*^ mice, however, suggesting that this parameter may not be a reliable indicator of the GIRK channel contribution to HRV in this frequency range.

Despite the fact that GIRK channels contribute to the inhibitory influence of CCh on ventricular myocytes, we did not see an obvious impact of ventricular GIRK channel ablation on QT or QTc intervals, at baseline or following CCh injection. It should be noted that previous attempts to model long-QT syndrome in mice have not reliably yielded mice with prolonged QT intervals^52^. For example, mice lacking *KvLQT1*, the gene associated with LQT1 in large mammals, do not consistently display prolonged QT intervals^52^. We also cannot exclude the possibility that the influence of ventricular GIRK channel becomes more relevant in pathological conditions such as myocardial infarction or sympathetic stress. We did see a surprising prolongation of the QTc interval after CCh administration in *Girk4*^−/−^ mice that was not seen in wild-type controls. While this observation may reflect a failure of the QT correction to adequately compensate for the impact of HR on QT interval or a limitation of this approach in mice under anesthesia^53,54^, the finding is also consistent with the possibility that CCh modulates another effector that exerts an opposing influence from the GIRK channel on the QT interval. Importantly, since MLC2VCre(+):*Girk1*^*fl/fl*^ and MLC2VCre(−):*Girk1*^*fl/fl*^ mice exhibited similar QT and QTc intervals at baseline and after CCh administration, the selective prolongation of QTc interval in *Girk4*^−/−^ mice presumably reflects an influence of GIRK channels outside of the ventricle.

I_KACh_-dependent signaling has been linked to a variety of atrial rhythm disorders^55–57^. Both genetic ablation of *Girk4* and pharmacological inhibition of GIRK channels can restore normal cardiac rhythm in multiple genetic models of supraventricular arrhythmia^55,56^. For example, genetic ablation of *Girk4* in mouse models of sick sinus syndrome and AV block reduces the number of ACh-induced arrhythmic episodes in these mice^56^. Additionally, fewer arrhythmic events were observed in *Girk4*^−/−^ mice treated with CCh as compared to wild-type controls (this study), and *Girk4*^−/−^ mice were resistant to pacing-induced AF^35^. Studies in human AF patients further implicate I_KACh_ in supraventricular arrhythmogenesis. Atrial myocytes from human AF patients displayed both a significant reduction in I_KACh_ expression and an attenuation of CCh-induced shortening of atrial APD^58^. CCh-induced I_KACh_ currents were also smaller in atrial myocytes from AF patients, whereas constitutive GIRK channel activity was elevated^58–60^. Remodeling involving I_KACh_ has also been observed in a “tachy-paced” dog model, where atrial tachycardia increased basal I_KACh_ channel activity^61^; this adaptation correlated with a decrease in ERP and an increase in AF duration. Collectively, these studies highlight the therapeutic potential associated with blocking or suppressing atrial I_KACh_ in multiple distinct atrial arrhythmias.

I_KACh_ antagonists have been proposed for use in the treatment of AF and other supraventricular rhythm disorders^56,57,62–64^. Recently, a novel family of direct-acting, small molecule activators and inhibitors of GIRK channels has been identified, members of which act selectively on GIRK1-containing GIRK channels such as I_KACh_^65–67^. While a direct-acting I_KACh_ antagonist could prove useful in the treatment of atrial rhythm disorders, suppressing GIRK channel activity in the ventricle could pose a significant risk. We did not, however, observe a difference in susceptibility to pacing-induced ventricular arrhythmias in either the constitutive or ventricle-specific GIRK channel ablation models, arguing that a loss of ventricular GIRK channel does not predispose to ventricular arrhythmias. However, given the noted differences in the ionic currents underlying ventricular repolarization in mice and humans^53^, further studies are needed to determine whether inhibition of the human ventricular GIRK channel is also without effect. Nonetheless, our data support the contention that targeting I_KACh_ for atrial arrhythmias, via with selective inhibitors or genetic approaches, may constitute a safe alternative to existing anti-arrhythmic therapies that predispose patients to fatal ventricular disorders^64^.

## Methods

### Animals

All experimental procedures involving mice were approved by the Institutional Animal Care and Use Committee of the University of Minnesota, and were conducted in accordance with guidelines set by the National Institute of Health. The generation of *Girk1*^−/−^, *Girk4*^−/−^, *Rgs6*^−/−^, and *Girk1*^*fl/fl*^ mice was described previously^19,20,45,46^. *Girk1*^*fl/fl*^ mice were crossed with MLC2VCre(+)^47^ mice to generate the MLC2VCre:*Girk1*^*fl/fl*^ line. B6.Cg-*Gt(ROSA)26Sor*^*tm14(CAG-tdTomato)Hze*^/J (Ai14-tdTomato) reporter mice were purchased from The Jackson Laboratory (Bar Harbor, ME) and crossed with MLC2VCre(+) mice. Mice were group-housed on a 12-h light/dark cycle, and given free access to food and water and were used for experiments at age 8–12 wk.

### Quantitative RT-PCR

Total RNA was isolated from freshly isolated atrial and ventricular tissue samples from adult C57BL/6J mice using the RNeasy fibrous tissue kit (Qiagen; Germantown, MD), according to manufacturer recommendations. Reverse transcription was performed using iScript™ cDNA Synthesis Kit (Bio-Rad Laboratories; Hercules, CA). Quantitative PCR was performed in a StepOnePlus Real Time PCR System (Applied Biosystems; Foster City, CA), with the Fast SYBR Green Master Mix (ThermoFisher Scientific; Waltham, MA). The following amplification program was used: 95 °C/20 s followed by 40 cycles of 95 °C/3 s, 60 °C/30 s. Intron-spanning primer pairs were as follows: M2R: 5′-GCCAGACTCCACCAGAT-3′ (forward) and 5′-CCATTGTTCGAGGAGTTAGTT-3′ (reverse); GIRK1: 5′-AAACTCACTCTCATGTTCCG-3′ (forward) and 5′-TCCAGTTCAAGTTGGTCAAG-3′ (reverse); GIRK4: 5′-GAGTTCGAAGTTGTGGTCATA-3′ (forward) and 5′-GCACCTCTGTATCCATGTAAG-3′ (reverse); RGS6: 5′-CTGACATTGTACAGTGGCTTAT-3′ (forward) and 5′-GAGAACATGGTCTGAGATTGG-3′ (reverse). The specificity of the GIRK1, GIRK4, and RGS6 reactions was assessed with a melting curve at the end of the program, and confirmed using samples from Girk1^−/−^, Girk4^−/−^, and Rgs6^−/−^ mice, respectively. Samples were tested in duplicate, and the average of replicates was used in the final data analysis. GAPDH (Mm_Gapd_2_SG QuantiTect primers; Qiagen) was used as internal control in each sample.

### Cell culture

SAN cells and ventricular myocytes were isolated as described^43,68^. Mice were injected intraperitoneally (i.p) with heparin (250 U) and then anesthetized with ketamine (100 mg/kg) and xylazine (10 mg/kg). To obtain SAN cells, hearts were excised into Tyrode’s solution (in mM): 140 NaCl, 5.4 KCl, 1.2 KH_2_PO4, 1.0 MgCl_2_, 1.8 CaCl_2_, 5.55 glucose, 5 HEPES, pH 7.4 with NaOH. SAN-containing tissue was excised into a modified Tyrode’s solution containing (in mM): 140 NaCl, 5.4 KCl, 1.2 KH_2_PO_4_, 0.2 CaCl_2_, 50 taurine, 18.5 glucose, 5 HEPES, 0.1% BSA, pH 6.9 with NaOH, with elastase (0.3 mg/mL, Worthington Biochemical Corp., Lakewood, NJ) and collagenase II (0.21 mg/mL; Sigma Aldrich, St. Louis, MO). SAN tissue was digested at 37  °C for 30 min and then washed three times in a solution containing (in mM): 100 L-glutamic acid/potassium salt, 10 L-aspartic acid/potassium salt, 25 KCl, 10 KH_2_PO_4_, 2 MgSO_4_, 20 taurine, 5 creatine, 0.5 EGTA, 20 glucose, 5 HEPES, 0.1% BSA, pH 7.2 with KOH. SAN tissue was then triturated and plated on laminin-coated coverslips (25 μg/mL) and used within 8 h.

Ventricular myocytes were extracted by excising hearts into ice-cold cell isolation buffer (in mM): 118 NaCl, 4.8 KCl, 25 HEPES, 1.2 KH_2_PO_4_, 1.2 MgSO_4_, 11 glucose, 30 taurine, 10 2,3-butanedione monoxime, pH 7.4 (with NaOH). The aorta was subsequently cannulated and hearts were then retrogradely perfused using a Langendorff apparatus with warm cell isolation buffer supplemented with collagenase II (620 U/mL; Worthington Biochemical Corp.). After a 10 min digestion, the heart was taken off the cannula and the atria were removed. The ventricles were cut into pieces and triturated. Samples were then transferred to a stop buffer consisting of cell isolation buffer supplemented with 2.5% BSA, 0.1 mM CaCl_2_, 5%FBS; where CaCl_2_ was added in three, 5 min increments to reach a final CaCl_2_ concentration of 1.6 mM. The cell suspension was then transferred to media consisting of M199, 0.2% BSA, 26.2 mM sodium bicarbonate, 25 mM HEPES, insulin-transferrin-sodium-selenite solution (1X), 5% FBS, penicillin (1000 U), streptomycin (1 mg), pH 7.4 (with NaOH); and plated on laminin-coated coverslips (25 μg/mL) and used within 8 h.

### Whole-cell electrophysiology

Coverslips containing adult SAN cells or ventricular myocytes were transferred to a perfusion chamber and electrophysiological recordings were conducted as described^45^. SAN cells were identified as thin, striated cells with capacitance values between 20–40 pF. Ventricular myocytes were identified as quiescent, rod-shaped cells with capacitance values ranging from 100–250 pF. Whole-cell currents and voltages were measured with hardware (Axopatch-700B amplifier, Digidata 1440A) and software (pCLAMP v. 10.4) from Molecular Devices (Sunnyvale, CA). All currents were low-pass filtered at 0.1 kHz, sampled at 5 kHz, and stored on computer hard disk for analysis. The liquid-junction potential, predicted to be −17 mV using JPCalc software (Molecular Devices), was not corrected. CCh-induced currents were measured at a holding potential of −70 mV. Borosilicate patch pipettes were filled with (in mM): 130 K-gluconate, 2 MgCl_2_, 1.1 EGTA, 5 HEPES, 2 Na_2_ATP, 5 phosphocreatine, and 0.3 Na-GTP, pH 7.2 (with KOH). Whole-cell access was achieved in a low-K^+^ bath solution consisting of (in mM): 130 NaCl, 5.4 KCl, 1 CaCl_2_, 1 MgCl_2_, 5.5 glucose, 5 HEPES/NaOH, pH 7.4. A high-K^+^ bath solution (+/−CCh) consisting of (in mM): 120 NaCl, 25 KCl, 1 CaCl_2_, 1 MgCl_2_, 5.5 glucose, 5 HEPES/NaOH, pH 7.4 was applied via ValveLink 8.2 rapid perfusion system (AutoMate Scientific, Berkeley, CA). Activation and deactivation time constants were extracted from appropriate regions of current traces, which were fit with a 1-term Boltzmann equation. Rheobase was evaluated in current-clamp mode in low-K^+^ bath solution by injecting increasing steps (5 pA/500 ms) of depolarizing current, starting at 0 pA, until an action potential spike was elicited. APD_20_, APD_50_, APD_70_, and APD_90_ were measured from the average of 10 spikes elicited by injecting a supra-threshold current (1 nA/5 ms) into the cell. The % change in APD and rheobase after CCh perfusion was measured as with the follows: ((baseline − post-CCh/baseline) × 100). Only those experiments in which the access resistances were stable and low (<20 MΩ) were included in the final analysis.

### Microscopy

Hearts were fixed with 4% paraformaldehyde in PBS at 4 °C for 16–24 h, transferred to a 10% sucrose/PBS solution at 4 °C for 24 h, and then transferred to a solution of 30% sucrose/PBS solution at 4 °C for at least 48 h. 20 μm thick slices were cut by cryostat at −20 °C and slices were mounted and stained with ProLong Gold Antifade reagent with DAPI (ThermoFisher Scientific; Waltham, MA). Fluorescent images were captured with an Olympus BX51W1 upright microscope with Disk Spinning Unit, using a 2X objective and digital CCD camera (C10600-10B, Hamamatsu Photonic System Corp; Bridgewater, NJ). Images were processed with Metamorph Advanced 7.7.7.0 (Molecular Devices).

### *In vivo* ECG recordings

Mice were anesthetized with 1.5% isoflurane supplemented with an air mixture of 40% O_2_/60% N_2_ to sustain stable HR. Platinum needle ECG electrodes connected to an IX-ECG-12 recorder (iWorx; Dover, NH) were placed subcutaneously into each limb. Baseline HR was recorded for 10 min, at which point CCh (1.0 mg/kg i.p.) was administered. HR and HRV analysis was performed on 1-min bins of stable recording from minutes 9–10 (baseline) and 15–16 min (post-CCh), using Kubios HRV 3.02 software^69^. Artifact detection/correction was utilized to reduce the impact of ectopic and skipped beats (either AV block or RR intervals which differ from the neighboring RR intervals by over 25%) on HR and HRV analysis. The frequency bands for HRV analysis were defined as very low frequency (VLF), <0.4; low frequency (LF), 0.4–1.5 Hz; and high frequency (HF), 1.5–5.0 Hz; using a 120 s window with a 50% overlap for Fast Fourier Fransform spectrum analysis using Welch’s periodogram method. Arrhythmic events were defined as instances of AV block (P wave with no corresponding QRS complex) and/or instances of tachycardia (at least 3–5 beats). PR and QT intervals were analyzed from the average of 100 cycles from either lead I or lead II during these segments, using LabScribe v3 software, only from recordings where the noise level was sufficiently low to clearly delineate the beginning and end of the indicated waveform deflections. The QT interval was corrected for changes in RR interval using the following formula: QTc = SQRT[QT/(RR/100)]^50^.

### *Ex vivo* burst pacing

Hearts were excised and submerged in cardioplegic solution (in mM): 280 glucose, 13.44 KCl, 12.6 NaHCO_3_, and 34 mannitol. The aorta was subsequently cannulated and retrogradely perfused with warm (37±1 °C), oxygenated Tyrode’s (in mM): 130 NaCl, 1.8 CaCl_2_, 4 KCl, 1.0 MgCl_2_, 1.2 NaH_2_PO_4_, 24 NaHCO_3_, 5.5 glucose, pH 7.4. Once hearts had been allowed to stabilize for 10 min, burst pacing at decreasing basic cycle lengths (BCL) (100 BCL down to 10 BCL, step-size 10) was applied 3 times to determine susceptibility to ventricular tachycardia (VT) or fibrillation (VF) at baseline via a stimulating electrode placed at the base of the left ventricle. Once the protocol was finished, 3 μM CCh was perfused into the hearts. After a 2–5 min stabilization period, the burst pacing protocol was run once again. Analysis was done using LabChart v7.3.8 software (ADInstruments; Colorado Springs, Colorado), where occurrences of VT lasting at least 7–8 beats, were tallied before and after CCh infusion.

### Statistical analysis

All data are presented as the mean ± SEM. Statistical analyses were performed using Prism 5 (GraphPad Software, Inc.; La Jolla, CA) software. Male and female mice were used in all studies, and groups were balanced for sex. Data were analyzed with Student’s two-tailed t-test (unpaired and paired), Fisher’s exact test, 1-way ANOVA, and 2-way ANOVA with repeated measures, as appropriate. For studies involving 2-way ANOVA analysis, only the interaction is reported if one was detected. The Bonferroni multiple comparison *post hoc* test was used following 1-way or 2-way ANOVA analyses, if justified. The level of significance was set at *P* < 0.05.

### Data availability

The datasets generated during and/or analyzed during the current study are available from the corresponding author on reasonable request.

## Electronic supplementary material

Supplementary Information
