# Supplementary material for: Expression and relevance of the G protein-gated K+ channel in the mouse ventricle
Source: Sci Rep. 2018 Jan 19;8:1192. doi: 10.1038/s41598-018-19719-x (PMC5775354; doi:10.1038/s41598-018-19719-x)
Supplement: Supplementary file 1 — Supplementary Information [file 41598_2018_19719_MOESM1_ESM.doc]

**SUPPLEMENTARY INFORMATION**

**Expression and relevance of the G protein-gated K^+^ channel in the mouse ventricle**

**Allison Anderson^1^, Kanchan Kulkarni^2^, Ezequiel Marron Fernandez de Velasco^1^, Nicholas Carlblom^1^, Zhilian Xia^1^, Atsushi Nakano^3^, Kirill A. Martemyanov^4^, Elena G. Tolkacheva^2^, and Kevin Wickman^1^,***

^1^ University of Minnesota, Department of Pharmacology, Minneapolis, MN 55455 USA

^2^ University of Minnesota, Department of Biomedical Engineering, Minneapolis, MN 55455

^3^ University of California, Department of Molecular, Cell & Developmental Biology, Los Angeles, CA 90095 USA

^4^ The Scripps Research Institute, Department of Neuroscience, Jupiter, FL 33458

* [wickm002@umn.edu](mailto:wickm002@umn.edu)

**Table 1. ECG waveform analysis for constitutive and ventricle-specific *Girk^–/–^* mice**

|  | Baseline | | | CCh | | |
| --- | --- | --- | --- | --- | --- | --- |
| Genotype | PR (ms) | QT (ms) | QTc (ms) | PR (ms) | QT (ms) | QTc (ms) |
| wild-type | 47.9±3.4 | 59.1±2.2 | 45.6±1.4 | 67.2±5.3^a^ | 99.5±4.8^a^ | 45.2±3.4 |
| *Girk4^–/–^* | 48.1±1.2 | 63.0±2.0 | 49.8±0.9 | 56.2±1.3^a,b^ | 80.8±3.6^a,c^ | 57.2±1.0^a,d^ |
| MLC2VCre(-) | 46.8±1.0 | 65.8±2.2 | 49.3±1.9 | 58.1±2.4^a^ | 100.3±5.4^a^ | 50.5±2.3 |
| MLC2VCre(+) | 47.2±1.1 | 63.0±2.9 | 49.0±1.6 | 55.3±1.4^a^ | 113.2±11.5^a^ | 53.3±3.3 |

Summary of PR, QT, and QTc intervals before and after CCh injection (1.0 mg/kg). Group sizes ranged from 5-11 mice per genotype. Two-way ANOVA analysis revealed an interaction between genotype and treatment (F_1,19_=28.8, *P*<0.001) for PR interval in wild-type and *Girk4^–/–^* mice. There was a main effect of treatment (F_1,22_=74.5, *P*<0.001) mice*,* but no main effect of genotype (F_1,22_=0.6, *P*=0.50) or interaction between genotype and treatment (F_1,22_=2.5, *P*=0.10) for the PR interval in MLC2VCre(+):*Girk1^fl/fl^* and MLC2VCre(-):*Girk1^fl/fl^* mice. Two-way ANOVA analysis revealed an interaction between genotype and treatment (F_1,14_=20.4, *P*<0.001) for QT interval in wild-type and *Girk4^–/–^* mice. There was a main effect of treatment (F_1,11_=66.9, *P*<0.001), but no main effect of genotype (F_1,11_=0.5, *P*=0.50) or interaction between genotype and treatment (F_1,11_=2.3, *P*=0.20) for the QT interval in MLC2VCre(+):*Girk1^fl/fl^* and MLC2VCre(-):*Girk1^fl/fl^* mice. Two-way ANOVA analysis revealed an interaction between genotype and treatment (F_1,14_=14.6, *P*<0.01) for QTc interval in wild-type and *Girk4^–/–^* mice. No main effect of treatment (F_1,11_=3.4, *P*=0.091) or genotype (F_1,11_=0.2, *P*=0.69), or interaction between genotype and treatment (F_1,11_=1.2, *P*=0.30), was found for QTc interval for MLC2VCre(+):*Girk1^fl/fl^* and MLC2VCre(-):*Girk1^fl/fl^* mice. Symbols: ^a^*P*<0.001 vs. baseline (within parameter); ^b,c,d^*P*<0.05, 0.01, and 0.001, respectively, vs. wild-type (CCh).

**
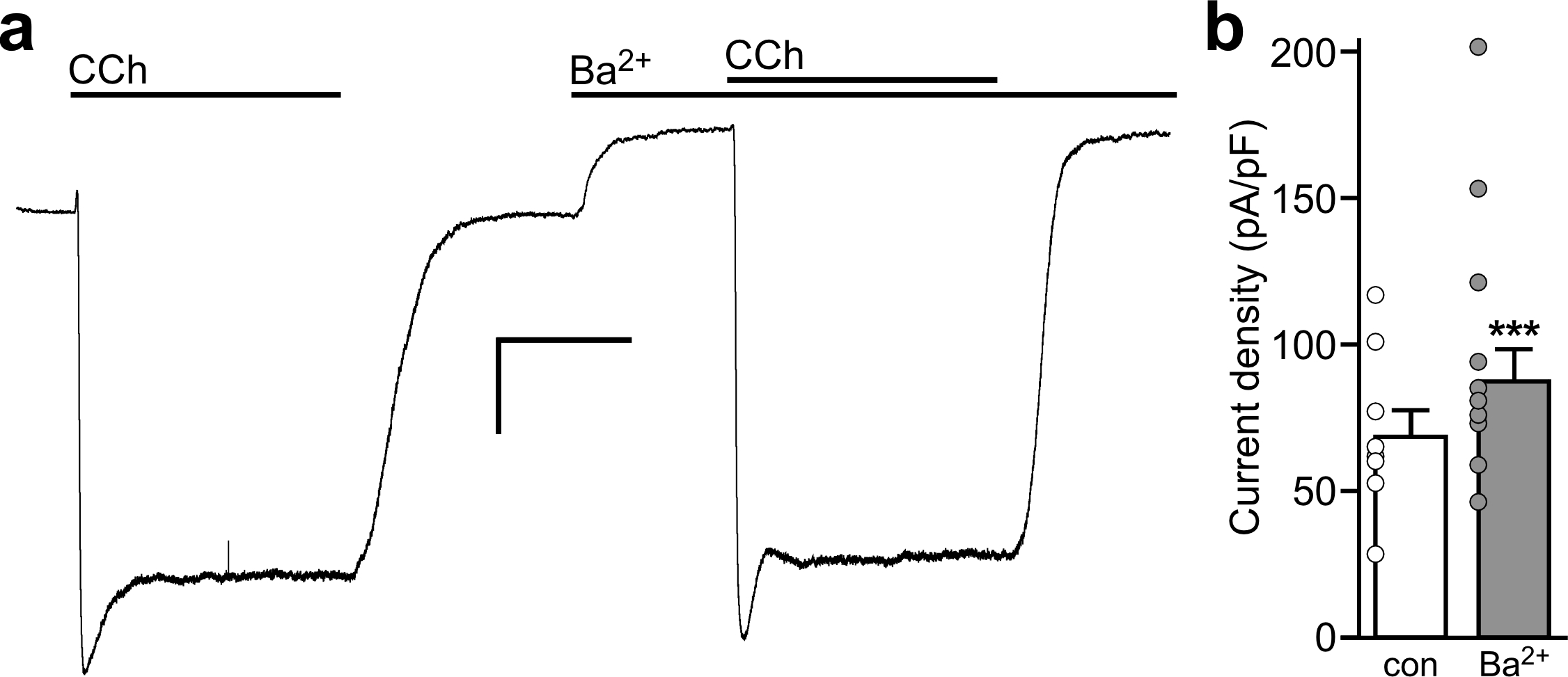
**

**Fig. S1. Impact of extracellular Ba^2+^ on CCh-induced GIRK current in mouse SAN cells**

**a)** Whole-cell current (V_hold_= -70 mV) evoked by carbachol (CCh, 10 μM) in an adult wild-type SAN cell, in the absence or presence of 5 μM extracellular Ba^2+^. Scale: 0.5 nA/10 s.

**b)** Summary of CCh-induced current density in adult wild-type SAN cells, measured in the absence (con) and presence (Ba^2+^) of 5 μM Ba^2+^ (*t*_8_=5.5, ****P*<0.001, n=9 cells/3 mice; paired Student’s t-test).


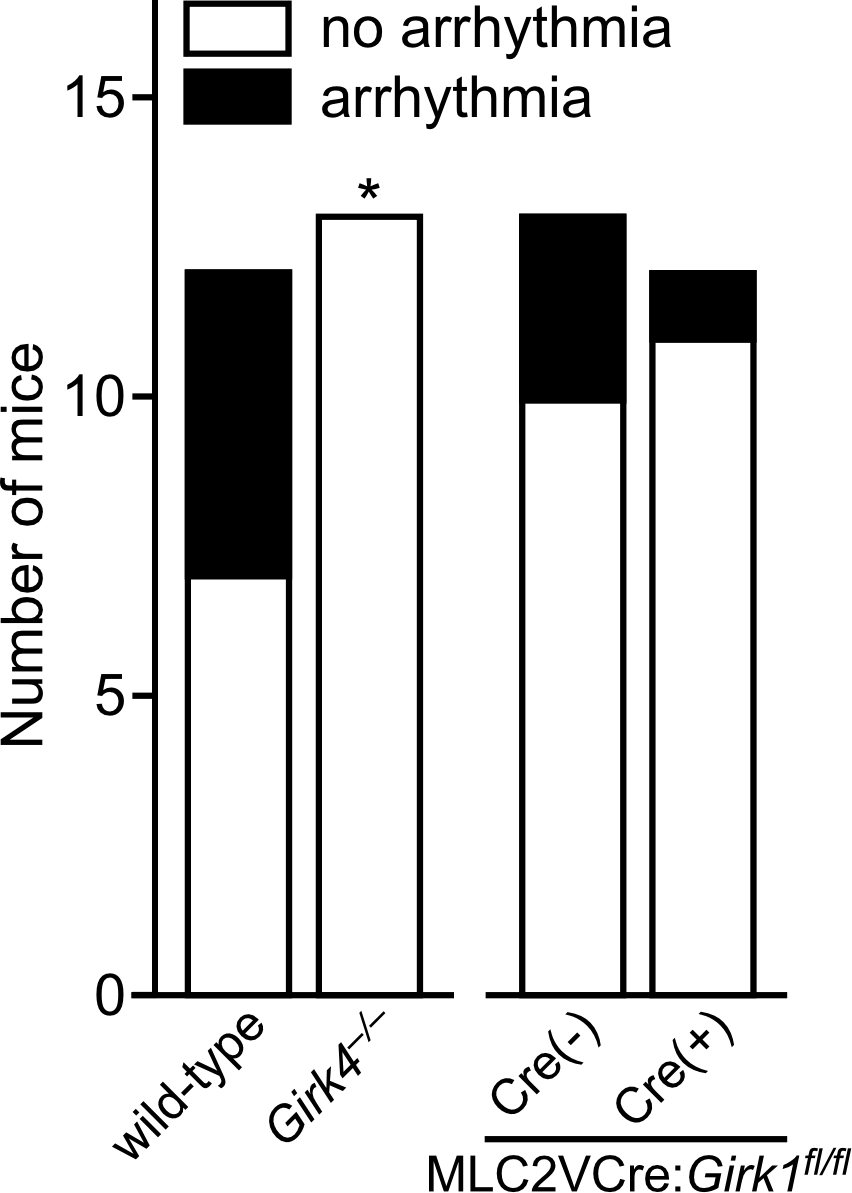


**Fig. S2. Impact of constitutive and ventricle-specific *Girk^–/–^* ablation on the frequency of arrhythmic events seen after CCh administration**

Summary of arrhythmic events (instances of AV block or tachycardia) following systemic CCh administration (1.0 mg/kg i.p.) in wild-type and *Girk4^–/–^* mice, as well as MLC2VCre(-):*Girk1^fl/fl^* and MLC2VCre(+):*Girk1^fl/fl^* mice. Fisher’s exact test revealed a significant difference (**P*<0.05) in the frequency of arrhythmic events observed in wild-type and *Girk4^–/–^* mice, but no difference (*P*=0.59) in the frequency of arrhythmic events between MLC2VCre(-):*Girk1^fl/fl^* and MLC2VCre(+):*Girk1^fl/fl^* mice.
